# Ets1 mediates sorafenib resistance by regulating mitochondrial ROS pathway in hepatocellular carcinoma

**DOI:** 10.1038/s41419-022-05022-1

**Published:** 2022-07-04

**Authors:** Kanchan Vishnoi, Rong Ke, Navin Viswakarma, Piush Srivastava, Sandeep Kumar, Subhasis Das, Sunil Kumar Singh, Daniel R. Principe, Ajay Rana, Basabi Rana

**Affiliations:** 1 Department of Surgery, Division of Surgical Oncology, University of Illinois at Chicago, Chicago, IL 60612 USA; 2 University of Illinois Hospital and Health Sciences System Cancer Center, University of Illinois at Chicago, Chicago, IL 60612 USA; 3 Jesse Brown VA Medical Center, Chicago, IL 60612 USA

**Keywords:** Cancer, Gastrointestinal cancer

## Abstract

The incidence and mortality of hepatocellular carcinoma (HCC) are on a rise in the Western countries including US, attributed mostly to late detection. Sorafenib has been the first-line FDA-approved drug for advanced unresectable HCC for almost a decade, but with limited efficacy due to the development of resistance. More recently, several other multi-kinase inhibitors (lenvatinib, cabozantinib, regorafenib), human monoclonal antibody (ramucirumab), and immune checkpoint inhibitors (nivolumab, pembrolizumab) have been approved as systemic therapies. Despite this, the median survival of patients is not significantly increased. Understanding of the molecular mechanism(s) that govern HCC resistance is critically needed to increase efficacy of current drugs and to develop more efficacious ones in the future. Our studies with sorafenib-resistant (soraR) HCC cells using transcription factor RT^2^ Profiler PCR Arrays revealed an increase in E26 transformation–specific-1 (Ets-1) transcription factor in all soraR cells. HCC TMA studies showed an increase in Ets-1 expression in advanced HCC compared to the normal livers. Overexpression or knocking down Ets-1 modulated sorafenib resistance-related epithelial–mesenchymal transition (EMT), migration, and cell survival. In addition, the soraR cells showed a significant reduction of mitochondrial damage and mitochondrial reactive oxygen species (mROS) generation, which were antagonized by knocking down Ets-1 expression. More in-depth analysis identified GPX-2 as a downstream mediator of Ets-1-induced sorafenib resistance, which was down-regulated by Ets-1 knockdown while other antioxidant pathway genes were not affected. Interestingly, knocking down GPX2 expression significantly increased sorafenib sensitivity in the soraR cells. Our studies indicate the activation of a novel Ets-1–GPX2 signaling axis in soraR cells, targeting which might successfully antagonize resistance and increase efficacy.

## Introduction

Liver cancer is the sixth most commonly occurring cancer and the third leading cause of cancer death worldwide [1] with a three times higher rate among males than females. Among the different types of primary liver cancers hepatocellular carcinoma (HCC), is the most common type originating from the hepatocytes under the settings of cirrhosis [2] and accounts for 70% of all liver cancers. The incidence of HCC is projected to increase in the coming decades due to its close association with obesity and metabolic syndrome [3]. About 40% of the HCC patients (very early stage) are eligible for curative treatment including resection, transplantation, or local ablation, and 20% (intermediate stage) for chemoembolization [4]. However, most HCC patients are diagnosed very late with advanced unresectable tumor, which makes them an unsuitable candidate for liver-directed therapy [5]. For the advanced HCCs, treatment with systemic drugs is the only option. Sorafenib, a multi-kinase inhibitor (MKI) is the first FDA-approved first-line systemic drug for the treatment of HCC [6] and is still in clinical use. Although patients respond initially to sorafenib treatment, most of them show disease progression within a few months [7], eventually acquiring sorafenib resistance and making the treatment unsuccessful. More recently, several other MKIs (lenvatinib, cabozantinib, regorafenib), antagonistic anti-VEGFR2 monoclonal antibody (ramucirumab), immune checkpoint inhibitors (nivolumab and pembrolizumab) have also been approved as first- or second-line options [8, 9]. Despite the availability of new drugs, the overall survival has not increased significantly, due to resistance [10]. Elucidating the mechanism of resistance and identifying novel targets involved in resistance is necessary for designing rational therapeutic strategies for long-term cure.

Cancer cells can develop therapeutic resistance using different molecular mechanisms which could be either intrinsic (primary resistance) or acquired (secondary resistance). The intrinsic resistance depends on genetic heterogeneity. On the other hand, acquired resistance is obtained during chemotherapy. Cancer cells may use different mechanisms to escape the cytotoxic effects of the drugs which include high expression and activity of drug transporters, increase in autophagy, invasion, migration, and survival of cancer stem cells [11]. Activation of oncogenic transcription factors (TFs) during the course of treatment can play a critical role by inducing the expression of genes involved in the manifestation of acquired chemoresistance [12]. Evidence suggests that aberrant and constitutive expression of oncogenic TFs in advanced cancers is correlated with chemoresistance and poor prognosis [13–15]. E26 transformation–specific-1 (Ets-1) is one such oncogenic TF, reported to contribute to the development and progression of a variety of cancers through transactivation or repression of various target genes [16–18]. Activation of Ets-1 in cancers is highly associated with chemoresistance [19–22] and linked to poor prognosis in ovarian cancer [23], breast cancer [24], and lung adenocarcinoma [25]. Ets-1 regulates several downstream genes including MMPs, integrin, and urokinase plasminogen activator (uPA) known for extracellular matrix degradation to promote cell migration and invasion [26, 27]. Moreover, higher expression of Ets-1 favors cancer cell growth and proliferation by regulating metabolism and oxidative stress [28–30].

To obtain an in-depth understanding of the mechanism of resistance in HCC, in the present study we focused on the mechanism of sorafenib resistance since this could be a common mechanism shared by other MKIs. Extensive studies over the last several years have linked multiple pathways mediating sorafenib resistance such as PI3K/AKT [31, 32], JAK-STAT [33], hypoxia [34], and more [7]. More recently, sorafenib combination therapies have shown some promising results in preclinical models [35–37]. Despite these advances, no effective drugs or combinations are currently available and advanced unresectable resistant HCCs are currently undruggable.

To obtain a more in-depth understanding of resistance, in the present study, we investigated the mechanism of sorafenib resistance using sorafenib-resistant (soraR) HCC cells, which revealed Ets-1 to be a critical regulator of this resistant pathway. Although Ets-1 has been linked with sorafenib resistance in earlier studies [38, 39], the downstream mechanism involved is not clearly defined. Our study revealed that Ets-1 expression is highly induced in the soraR HCC cells and in advanced HCC tumors. In the HCC cells, Ets-1 promoted migration, increased expression of EMT markers, reduced mitochondrial ROS (mROS), and increased apoptosis resistance. Interestingly, Ets-1-induced apoptosis resistance seems to be mediated by antioxidant protein GPX2. Taken together, our study reveals Ets-1 and GPX2 as key targets in sorafenib resistance and rationalizes the need to develop targeted drugs for antagonizing this axis to overcome HCC resistance.

## Materials and methods

### Reagents and antibodies

DMEM, DMEM/F12, MEM and Opti-MEM media, TRIzol and Lipofectamine 2000, MitoSOX, and CellEvent Caspase-3/7 green detection kit were purchased from Invitrogen (Carlsbad, CA); Sorafenib from Enzo Life Sciences, Inc. (Farmingdale, NY), Ultra-low attachment plates and MTT from Millipore Sigma (Burlington, MA), FITC Annexin V Apoptosis Detection Kit from BD Biosciences (San Jose, CA), JC-1 dye, Doxycycline Hydrochloride, and Transwell inserts from Thermo-Fisher Scientific (Waltham MA). The antibodies utilized were obtained from the following sources: Ets-1 (Cell Signaling Technology, #14069), E-cadherin (BD Biosciences, #610181, N-cadherin (BD Biosciences, #610920), Vimentin (Cell Signaling Technology, #5741), Snail (Cell Signaling Technology, #3895), Slug (Cell Signaling Technology, #9585), Zeb2 (Cell Signaling Technology, #97885), GAPDH (Ambion, #AM4300), PARP (Cell Signaling Technology, #9542), Cleaved Caspase 3 (Cell Signaling Technology, #9664), Myc-tag (Cell Signaling Technology, #2276), GPX2 (Abcam, #137431), Cytochrome C (Cell Signaling Technology, #11940), COX IV (Abcam, #14744). RT2 profiler PCR Array Human Transcription Factors (PAHS-075Z) was from Qiagen (Germantown, MD).

### Cell culture and creation of soraR cells

HCC cells (Hep3B, HepG2) were obtained from ATCC and maintained in MEM media supplemented with 10% FBS, 1% Pen/Strep, 1% HEPES, 1% sodium pyruvate, and 1% non-essential amino acids. Huh7 cells were obtained as described [40, 41] and maintained in DMEM/F12 media with 10% FBS and 1% Pen/Strep. All cells were authenticated using a short tandem repeat analysis. The soraR HCC cells were established by prolonged exposure to increasing concentrations of sorafenib. Briefly, cells plated in 100 mm dishes were allowed to reach ~80% confluence before being treated with the starting concentration of 2 µM sorafenib. They were maintained with the same sorafenib dose until they started growing normally after initial cell death. They were treated stepwise with escalating doses of sorafenib (from 2, 4, and 6 µM) in a similar manner until the cells achieved resistance to 6 µM sorafenib dose. Thereafter, the resistant cells were named Huh7-soraR, Hep3B-soraR, and HepG2-soraR to distinguish them from the sorafenib-sensitive (or naive) counterparts Huh7, Hep3B, and HepG2 respectively.

### MTT assay

To check the degree of sorafenib resistance, naive and soraR cells were treated with different concentrations of sorafenib, and the cell viability was assessed by performing the MTT assay as described [42] with modifications. Briefly, 5000 cells plated in triplicate in 96 well plates were treated with different concentrations of sorafenib ranging from 2 to 10 µM for 72 h. At the end of treatment, cells were incubated with 0.5 mg/ml MTT solution (in PBS) for 2 h at 37 °C, followed by incubation with DMSO for an additional 15 min at 37 °C. Thereafter, the absorbance was measured at 570 nm in a microtiter plate reader.

### Small interference RNA (siRNA)

Smart pool siRNA against hETS1 (cat # L-003887-00-0005) and a set of four individual siRNA against hGPX2 (cat # LQ-011675-00-0005) were purchased from Dharmacon (Lafayette, CO). A negative control siRNA (Ambion Inc., Austin, TX) was used as the control siRNA. Transient transfection using siRNA was performed using Lipofectamine 2000 reagent as per the manufacturer’s instructions and as described previously [43]. Briefly, 4 × 10^5^ cells plated in 35 mm plates were transfected with 50 nM of control or target siRNA for 24 h and were allowed to recover in complete media for another 24 h. Thereafter, the transfected cells were treated with either DMSO or sorafenib for 24–48 h and then analyzed by different assays.

### Transwell migration assay

The effect of sorafenib resistance on the migration of HCC cells was examined by transwell migration assays. Briefly, naive and soraR cells were trypsinized and 5 × 10^4^ cells/well were plated in the upper chamber of a transwell plate. To determine the role of Ets-1 on migration, soraR cells were transfected first with control- or Ets-1 siRNA for 48 h and then plated in transwell plates, as above. The cells in transwell plates were incubated at 37 °C and allowed to migrate for 48 h. At the time of harvest, they were fixed using 4% paraformaldehyde for 10 min, washed twice with PBS, and permeabilized using methanol for 20 min. The cells were then washed and stained using a 0.05% crystal violet staining solution for 15 min. The cells from the upper surface of the inserts were removed using a cotton swab, the inserts were air-dried, and images were acquired using NIS-Elements imaging software in Nikon Eclipse Ti Microscope. For quantitation, cells were counted in four different fields and plotted as bar graphs.

### Sphere formation assay

To determine the sphere formation efficiency of naive and soraR cells, 0.5 × 10^5^ of each cell type were plated in the 6-well ultra-low attachment plates in serum-free media with 1% pen–strep, 1× B27 (Thermo-Fisher Scientific, Waltham, MA, # 12587010), 1 x N2 supplement (Thermo-Fisher scientific # 17502048,), 20 ng/ml EGF (Peprotech, Cranbury, NJ, # AF-100-15) and 10 ng/ml bFGF (Peprotech, # 100-18B) as described earlier with a little modification [44]. The spheres were allowed to grow for 7 days, and phase-contrast images were taken using NIS-Elements imaging software in Nikon Eclipse Ti Microscope. They were then harvested and analyzed by qPCR.

### Estimation of apoptosis and mitochondrial damage

Apoptosis assays were performed using FITC Annexin V Apoptosis Detection Kit (BD Biosciences), and JC-1 assays (to detect mitochondrial damage) were performed using JC-1 dye by flow cytometry, as described previously [45]. Briefly, the control and treated cells were harvested by trypsinization and distributed equally into two parts for the detection of apoptosis and mitochondrial damage. The cells for apoptosis assay were stained with Annexin V and Propidium Iodide and those for JC-1 assay were incubated with JC-1 dye and the respective assays were performed using a Gallios flow cytometer (Beckman Coulter). The data were analyzed using the FlowJo software.

### Detection of mROS

To estimate the levels of mROS, cells were harvested and incubated with 5 µM MitoSOX prepared in 1 ml of pre-warmed media. The cells were then incubated for 15 min at 37 °C protected from the light. While harvesting, the cells were washed twice with PBS, re-suspended in 500 µl of PBS, and data were acquired using a Gallios flow cytometer. The data were analyzed using the FlowJo software.

### Caspase 3/7 assay

Caspase 3/7 activity was determined using CellEvent Caspase-3/7 green detection kit (from Invitrogen) as per the manufacturer’s instructions [46]. Briefly, 1 × 10^6^ cells treated with vehicle or sorafenib were suspended in 1 ml of PBS. 1 µl of CellEvent Caspase-3/7 green detection reagent was added to each sample to make a final concentration of the reagent to 500 nM. The cells were then incubated for 30 min at 37 °C protected from light, followed by data acquisition using Gallios flow cytometer and analysis using FlowJo software.

### RT2 Profiler PCR Array

Total RNA extracted from naive and soraR HCC cells using TRIzol reagent were reverse transcribed to cDNA and subjected to Real-Time PCR analysis with human transcription factors PCR Array (PAHS-075Z, Qiagen) on StepOne Plus machine as described earlier [42]. Fold changes in mRNA expression in soraR compared to naive cells were analyzed using Qiagen RT2 Profiler PCR Array Data Analysis Webportal (https://geneglobe.qiagen.com/us/analyze).

### Quantitative polymerase chain reaction (qPCR) analysis

Total RNA was extracted from the naive and soraR cells under various treatment conditions as above. cDNA was synthesized using Superscript III First-Strand Synthesis System kit (Invitrogen, Carlsbad, CA) as per the manufacturer’s instructions. qPCR analysis was performed as described [42] using SYBR Green PCR Master Mix (Applied Biosystems) in ABI StepOnePlus machine (Applied Biosystems). The PCR cycling condition was set as: an initial denaturation step at 95 °C for 2 min, 40 cycles at 95 °C for 15 s, 60 °C for 1 min finally subjecting to melting temperature to check the amplification curve. The relative changes in gene expression were estimated using the 2–ΔΔCt method using 18S rRNA as a housekeeping gene. The lists of primers used are included in Supplementary Table S1.

### Immunocytochemistry and immunohistochemistry

For immunocytochemistry, 1 × 10^6^ cells were plated in 6-well plates with a coverslip. The next day, the coverslips were transferred to a 12-well plate and were fixed using 4% paraformaldehyde for 20 min at room temperature, washed, and permeabilized using 1% Triton X-100 in PBS for 10 min. Blocking was performed using 5% BSA solution in PBS for 2 h followed by incubation with the primary antibody in 5% BSA–PBS at 4 °C overnight. The cells were then washed, incubated with the secondary antibody for 1 h at room temperature, and developed using DAB (Vector Laboratories, USA). The images were captured using NIS-Elements imaging software in Nikon Eclipse Ti Microscope. The immunohistochemistry staining was done as described previously with minor modifications [47]. In brief, the liver cancer tissue microarray (US Biomax, # T031b) was rehydrated in a decreasing alcohol gradient and antigen retrieval was performed using sodium citrate buffer in the decloaking chamber. The endogenous peroxidase activity was quenched by incubating the tissue with BLOXALL endogenous blocking solution (Vector Laboratories, USA) followed by blocking with 5% goat serum for 1 h. The slides were incubated with anti-Ets-1 (Abcam 1:100) primary antibody overnight at 4 °C. The slides were washed with PBST (PBS containing 0.1% Triton X), incubated with a secondary HRP antibody (Vector Laboratories, USA), and developed using DAB. The nuclei were counterstained with hematoxylin and mounted. Images were acquired using Leica Aperio Scanning and analyzed using Aperio imageScope at ×40 magnification.

### Creation of lentiviral Ets-1 expression vector

The inducible Ets-1 lentiviral vector was created using gateway cloning as described earlier [48]. Briefly, human Ets-1 cDNA was amplified from pDONR223_ETS1_WT (Addgene # 82118) using primers attB1-hEts-1: GGGGACAAGTTTGTACAAAAAAGCAGGCTACCATGAAGGCGGCCGTCGATCTCAAGCCGACTCTCAC and attB2-Myc-hEts-1 GGGGACCACTTTGTACAAGAAAGCTGGGTCTATTACAGATCCTCTTCTGAGATGAGTTTTTGTTCCTCGTCGGCATCTGGCTTGACGTCCAGCATGGCGTGCAGCTCC and cloned into pDONR221 vector (Life Technologies, USA, # 12536017) using BP Clonase (Life technologies, USA # 11789100), to make the entry clone. The LR recombination reaction was performed with the entry clone using LR Clonase (Life technologies, USA, # 11791020) to generate the doxycycline-inducible Ets-1 lentiviral vector in pLIX_403 backbone (Addgene # 41395).

### Viral production and transduction to create the Ets-1 stable cell lines

Lentiviral particles containing myc-tagged human Ets-1 were produced as described previously [47]. Briefly, HEK 293FT cells (Life Technologies, USA) were co-transfected with psPAX2, pMD2G packaging plasmids, and pLIX-403-Ets-1 WT plasmid using Lipofectamine 2000 reagent. The supernatant containing the lentiviral particles was collected. The transduction efficiency of the lentiviral particles was checked by transducing Huh7 cells with different volumes of the lentiviral supernatant and by performing western blots for Ets-1. Accordingly, the Huh7 and Hep3B cells were transduced with an optimal volume of lentiviral supernatant and stable cell lines were selected using puromycin (4 µg/ml for Huh7 cells and 2 µg/ml for Hep3B cells). The stable cells were treated with Doxycycline (1 µg/ml) to induce ectopic Ets-1 expression.

### Western blot analysis

Western blot analysis was performed following procedures described earlier [45]. Briefly, equal amounts of cell extracts were separated by SDS-PAGE, transferred to PVDF membranes, and subjected to Western blot analysis utilizing various antibodies. To detect the cytochrome c release from the mitochondria to the cytoplasm following sorafenib treatment, the cytoplasmic and mitochondrial proteins were fractionated from the cells as described [45]. The cytoplasmic and nuclear extracts were then subjected to western blot analysis to detect cytochrome c release.

### Statistical analyses

Student’s *t* test was performed and expressed as *p ≤ 0.05, **p ≤ 0.01, ***p ≤ 0.001, ****p ≤ 0.0001, ns: *p* > 0.05 not significant.

## Results

### Creation and characterization of soraR HCC cells

**Fig. 1:**
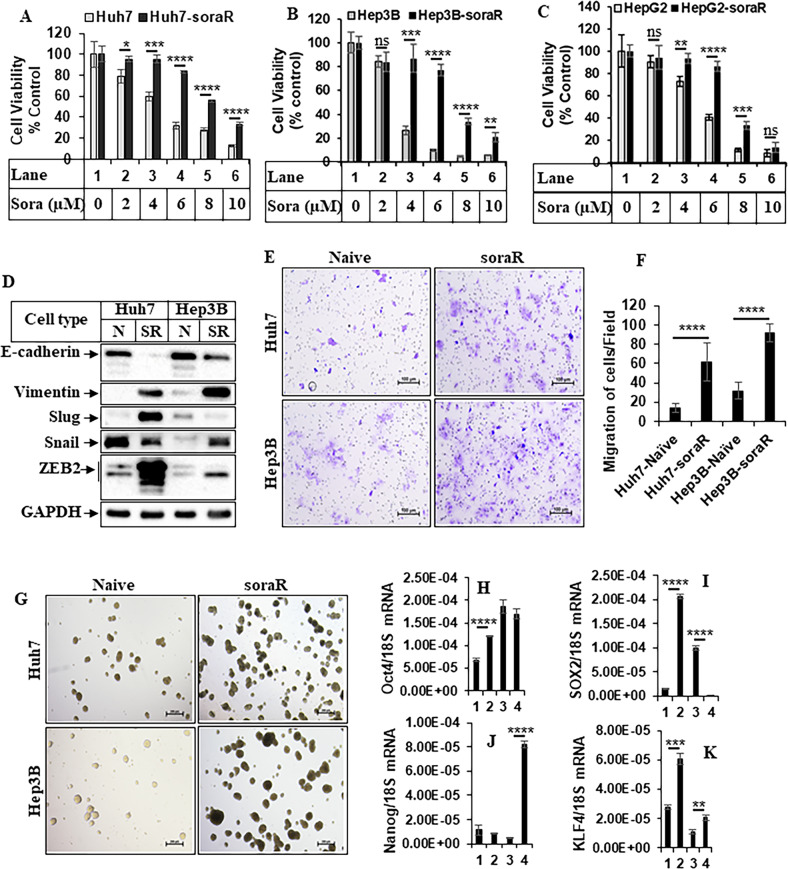
Characterization of soraR HCC cells. **A** Huh7, **B** Hep3B, and **C** HepG2 naive and sorafenib-resistant (soraR) cells were treated with increasing concentrations of sorafenib and subjected to MTT assay after 72 h. The values were expressed as % control considering DMSO-treated samples as 100%. Each assay was performed in triplicate, and each experiment was repeated at least two times. The data represent the mean ± S.D of three independent assays. **D** Equal amounts of total protein from naive (N) and soraR (SR) HCC cells were analyzed by western blots with the indicated antibodies. **E** Naive and soraR cells plated in transwell plates were allowed to migrate for 48 h and images were captured using NIS-Elements imaging software in Nikon Eclipse Ti Microscope. Scale bar, 100 µm. For quantitation, cells were counted in four different fields and plotted as bar graphs (**F**). The data represent the mean ± S.D. of two independent experiments. **G** Naive and soraR cells were plated in ultra-low attachment plates to form spheres, and the spheres were photographed on day 7 using NIS-Elements imaging software in a Nikon Eclipse Ti microscope. Scale bar, 200 µm. **H**–**K** RNA extracted from naive and soraR spheres as described in **G** were analyzed by qPCR with the indicated genes. Lane 1: Huh7-N, Lane 2: Huh7-SR, Lane 3: Hep3B-N, Lane 4: Hep3B-SR. The data represent the mean ± S.D. of three independent qPCR reactions. Significant differences were determined by *t* test and indicated as: ns, *p* > 0.05; *p ≤ 0.05; **p ≤ 0.01; ***p ≤ 0.001; ****p ≤ 0.0001.

To understand the detailed mechanism mediating resistance to standard therapy in HCC, soraR cells were generated by prolonged exposure of HCC cells to increasing doses of sorafenib up to a maximum of 6 µM. These cells were named Huh7-soraR, Hep3B-soraR, and HepG2-soraR to distinguish them from their sorafenib-sensitive (naive) counterparts (Huh7, Hep3B, HepG2, respectively). Treatment with increasing doses of sorafenib showed all soraR cells to be resistant to sorafenib at least up to a concentration of 6 µM (Fig. 1A–C), which is reported to be a clinically relevant dose of sorafenib [49]. Thus, this concentration of sorafenib was utilized in the following studies. A comparison of the expression of various EMT markers between the naive and soraR cells showed an increase in EMT phenotype, which include reduced E-cadherin and increased Vimentin and Zeb2 expression levels (Fig. 1D). While slug and Snail expression levels were mutually exclusive, at least one of them was induced in each soraR cell. In addition, transwell migration assays showed a higher migratory potential of the soraR cells compared to naive (Fig. 1E, F). As drug resistance is the main property of the cancer stem cells (CSCs) that can be assessed by sphere formation, the sphere-forming efficiency between naive and soraR cells was examined next. The soraR cells displayed higher efficiency of sphere formation as compared to their naive counterparts (Fig. 1G). The transcript levels of the stemness genes were also analyzed by qPCR (Fig. 1H–K) which showed a differential pattern of upregulation. The transcript levels of Oct-4, Sox-2, and KLF4 were significantly elevated in the Huh7-soraR cells, while Nanog and KLF4 were increased in Hep3B-soraR cells.

### SoraR HCC cells are resistant to apoptosis, mitochondrial damage, and mROS production

**Fig. 2:**
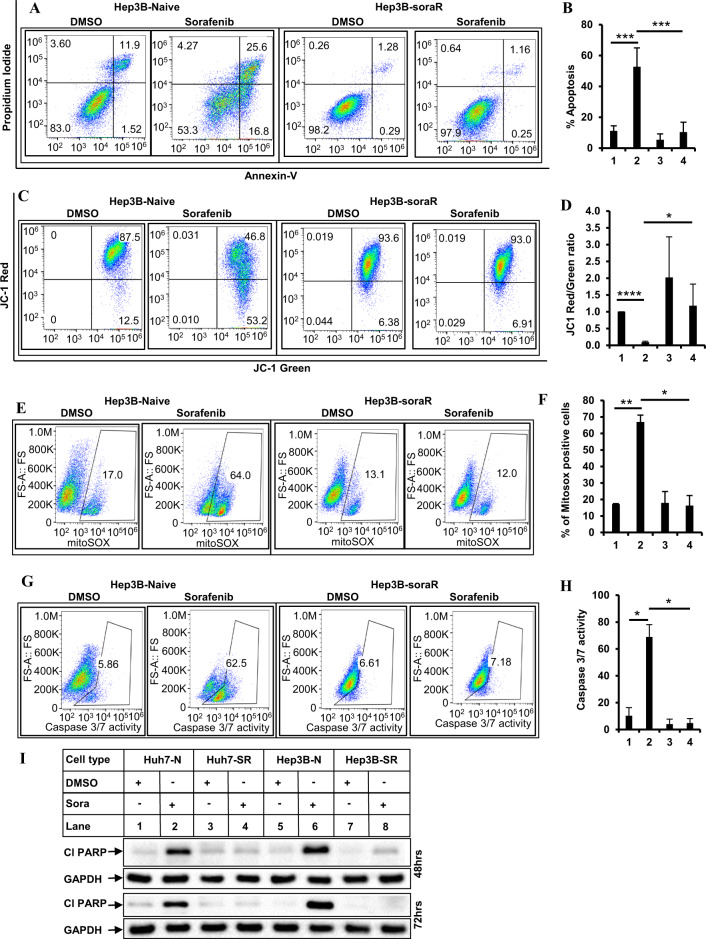
soraR cells are resistant to sorafenib-induced apoptosis and mitochondrial damage. **A** Representative images of flow-cytometric detection of apoptosis in naive and soraR Hep3B cells treated with DMSO or sorafenib (6 µM) for 72 h. Cells were harvested and analyzed for apoptosis using FITC Annexin V Apoptosis Detection Kit. The bar graphs in **B** represent the % of total apoptosis under different treatment conditions. **C** Hep3B-naive and soraR cells treated as in **A** were analyzed to detect changes in mitochondrial membrane potential after staining with JC-1 dye. The bar graphs in **D** represent the ratio of JC-1 Red/JC-1 green. **E** Hep3B-naive and soraR cells treated with DMSO or sorafenib (6 µM) for 24 h were incubated with 5 µM MitoSOX, followed by flow cytometry to detect mitochondrial ROS. The bar graphs on the right (**F**) represent the % of mitosox-positive cells. **G** Hep3B-naive and soraR cells treated as in **A** were incubated with CellEvent Caspase-3/7 green detection reagent and subjected to flow cytometry to detect caspase 3/7 activity. The bar graphs on the right (**H**) represent changes in caspase 3/7 activity under different treatment conditions. The data (in **B**, **D**, **F**, **H**) represent the mean ± S.D. of two to four independent experiments. Hep3B-N cells were treated with DMSO (lane 1), or sorafenib (lane 2); Hep3B-SR cells were treated with DMSO (lane 3), or sorafenib (lane 4). Significant differences were determined by *t* test and indicated as: *p ≤ 0.05; **p ≤ 0.01; ***p ≤ 0.001; ****p ≤ 0.0001. **I** Naive and soraR HCC cells were treated with DMSO or sorafenib (6 µM) for 48 and 72 h and analyzed by western blots with antibodies against PARP or GAPDH. PARP poly (ADP-ribose) polymerase, Cl PARP cleaved PARP.

To determine any changes in apoptotic potential, naive and soraR cells treated with sorafenib were subjected to flow cytometry. These showed that although sorafenib can induce significant apoptosis in the naive cells, the soraR cells were highly resistant (Fig. 2A, B and Supplementary Fig. S1A, B). Similarly, JC-1 staining showed increased mitochondrial damage with sorafenib in the naive cells, and less in the soraR cells (Figs. 2C, D and S1C, D). Interestingly, the soraR cells showed increased expression of at least one Bcl-2 family pro-survival member and were sensitized to sorafenib by Bcl-xL inhibitor ABT-263 (navitoclax) or knocking down Bcl-xL expression (data not shown). These were similar to the observations reported earlier by other investigators [50]. Since increased mROS is known to promote apoptosis following chemotherapeutic drugs, we also compared the levels of mROS production between the naive and soraR cells. As shown in Figs. 2E, F and S1E, F, there was a significant reduction in the generation of mROS in the soraR cells. The activation of caspase 3/7 and cleavage of PARP was substantially reduced in the soraR cells (Figs. 2G–I and S1G, H). Taken together, these suggest that sorafenib resistance leads to inhibition of apoptosis induction, which might be linked with inhibition of mROS production.

### Changes of gene expression profiles in soraR cells and induction of Ets-1 in soraR

**Fig. 3:**
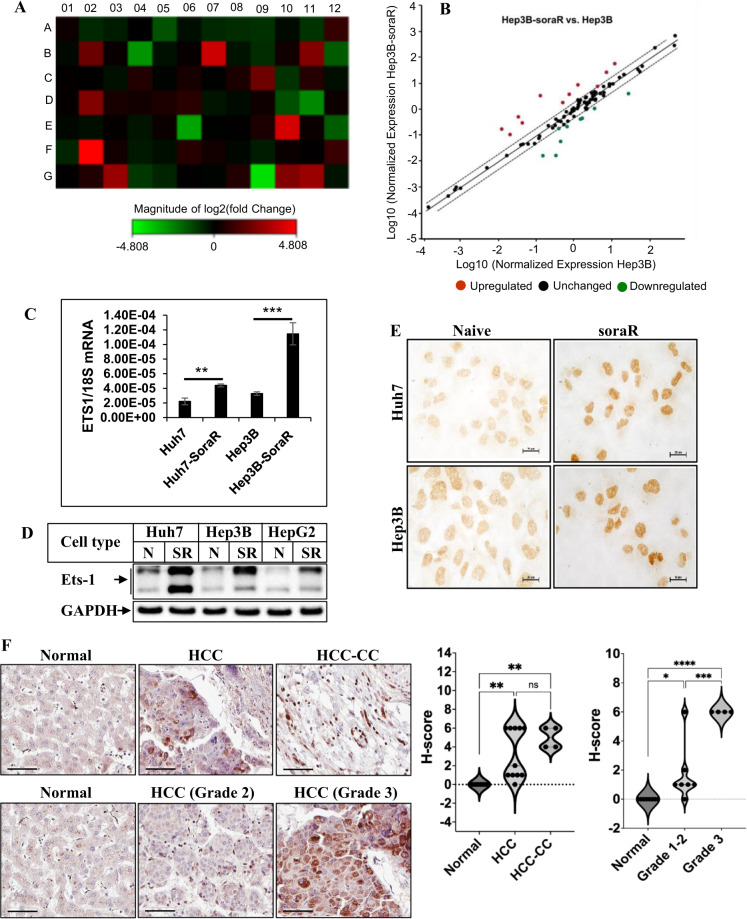
Transcription factor Ets-1 expression is induced in soraR cells. **A** Total RNA extracted from Hep3B-naive and soraR cells was subjected to cDNA synthesis and analyzed with human transcription factors PCR Array (PAHS-075Z). Figure in **A** shows the heat map with the upregulated genes marked in red and the downregulated genes marked in green. **B** shows the scatter plot of expression levels of 84 genes. Red dots indicate genes upregulated and the green dots indicate the genes downregulated. The central line indicates genes that were unchanged (black dots) with dotted lines indicating the selected twofold regulation cut-of. **C** Changes in the expression of ETS-1 gene in Huh7 and Hep3B (naive and soraR) cells were analyzed by qPCR. The data represent the mean ± S.D. of 3 independent PCR reactions, and the experiment was repeated three times. **D** Equal amounts of total protein from 3 sets of naive (N) and soraR (SR) HCC cells were analyzed by western blots with antibodies against Ets-1 and GAPDH. **E** Immunocytochemistry showing differences in expression of Ets-1 in naive and soraR cells. The images were captured using the NIS-Elements imaging software in Nikon Eclipse Ti Microscope. Scale bar, 20 µm. **F** Representative images of immunohistochemistry of Ets-1 using liver cancer tissue microarray (US Biomax, # T031b). Scale bar, 60 µm. The Ets-1 staining intensities in the TMA were scored and plotted to the right. Statistical significance was determined by Tukey’s multiple comparisons test. *p < 0.05, **p < 0.01, ***p < 0.001, ****p < 0.0001, ns: *p* > 0.05 not significant. HCC-CC hepatocholangiocarcinoma.

Based on the above data, our next goal was to identify in an unbiased approach the potential mediator(s) promoting cell survival in sorafenib resistance. Since the Bcl-2 family proteins (which were also induced in our soraR cells) are regulated transcriptionally [51, 52], we hypothesized that transcription factors might be involved in mediating this resistance. RNA extracted from naive and soraR cells were analyzed by RT^2^ Profiler PCR Transcription Factor Arrays (PAHS-075Z from Qiagen) as per the manufacturer’s instructions and as described [42]. These revealed increased expression levels of the transcription factor (TF) ETS1 in Hep3B-soraR cells (Figs. 3A, B and S2A) and Huh7-soraR (Fig. S2B, C) cells. Although a few other transcription factors were also induced, they did not show uniform induction in all soraR cells (Fig. S2D). The expression of Ets-1 was further validated at the transcript and the protein levels by qPCR (Fig. 3C) and western blots (Fig. 3D) respectively, which showed that Ets-1 was induced significantly in all soraR cells. Immunocytochemistry staining of Ets-1 revealed a higher nuclear expression of Ets-1 in the soraR cells (Fig. 3E). Furthermore, Immunohistochemistry (IHC) staining of HCC tissue microarrays showed an increase in Ets-1 expression in advanced stages of cancer (Fig. 3F).

### Modulation of Ets-1 expression mediates EMT phenotype

**Fig. 4:**
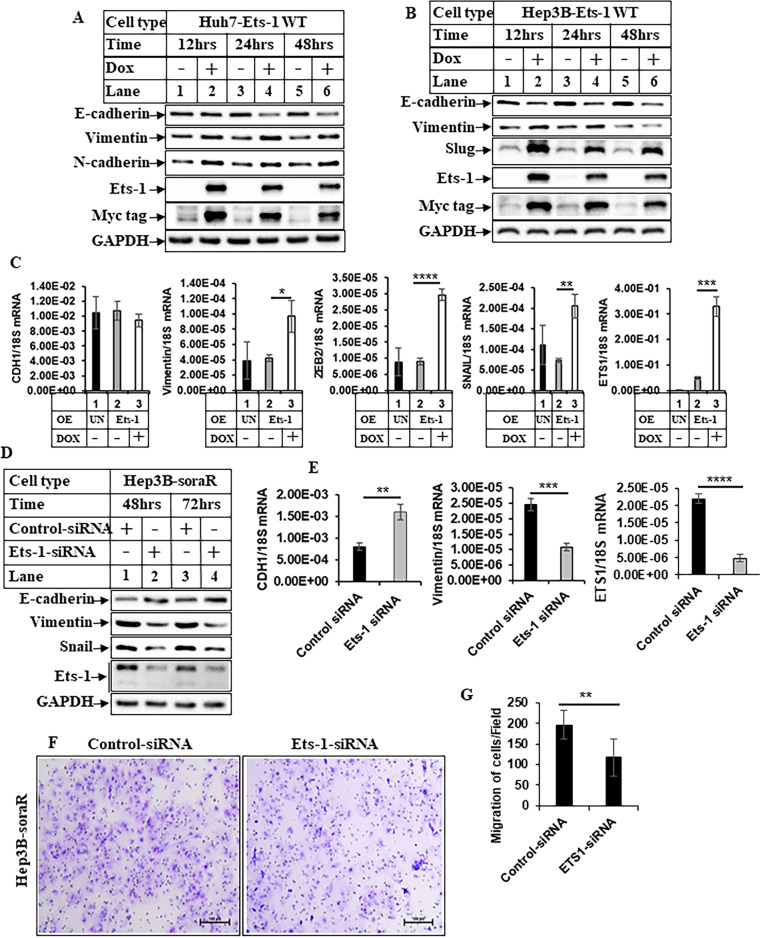
Ets-1 regulates epithelial–mesenchymal transition (EMT) in soraR cells. **A** Huh7 naive and **B** Hep3B naive cells stably overexpressing Ets1-WT (Huh7-Ets-1 WT and Hep3B-Ets-1 WT respectively) were treated with (+) or without (−) 1 µg/ml Doxycycline (DOX) to induce ectopic Ets-1 expression in low serum (1% FBS) media. The cells were harvested at various time points and analyzed by western blots with the indicated antibodies. **C** Subconfluent Hep3B naive cells were transiently transfected with pLIX-403-Ets-1 and treated without DOX (lane 2) or with DOX (lane 3) for 48 h followed by RNA extraction and qPCR analysis of the indicated EMT genes. UN- indicates untransfected control and OE- indicates overexpression. **D** Hep3B-soraR cells transiently transfected with control- or Ets-1-siRNA for 48 and 72 h were analyzed by western blots. **E** Hep3B-soraR cells transfected as in **D** for 72 h were analyzed by qPCR for EMT genes. The data in **C**, **E** represent the mean ± S.D. of three independent PCR reactions. **F** Hep3B-soraR cells transfected with control- or Ets-1-siRNA were subjected to transwell migration assay for 48 h as in Fig. 1E. Scale bar, 100 µm. For quantitation, cells were counted in four different fields and plotted as bar graphs (**G**). The data represent the mean ± S.D. of two independent experiments. Significant differences were determined by *t* test and indicated as: *p ≤ 0.05; **p ≤ 0.01; ***p ≤ 0.001; ****p ≤ 0.0001.

Ets-1 has been linked with therapeutic resistance earlier [53, 54]. In fact, recent studies have also shown Ets-1 to be involved in mediating sorafenib resistance [38, 39]. Despite these, the downstream mechanism and biological events mediated by Ets-1-induced resistance are still unclear and need to be elucidated. To determine whether Ets-1 regulates the pro-oncogenic phenotypes observed in the soraR cells, we created stable Doxycycline (DOX)-inducible Ets-1-overexpressing Huh7 and Hep3B (naive) cells following procedures described earlier [45]. These cells were termed Huh7-Ets1-WT and Hep3B-Ets1-WT cells. Interestingly, overexpression of Ets-1 in the naive cells showed an increase in EMT phenotype (Fig. 4A, B). In addition, transient overexpression of Ets-1 also regulated the EMT-specific genes as shown by qPCR analysis (Fig. 4C). There was, however, no significant difference in sphere formation with Ets-1 induction (data not shown). As a complementary approach, endogenous Ets-1 was knocked down by siRNA in the soraR cells. These showed that knocking down endogenous Ets-1 reduced EMT phenotype of soraR cells (Figs. 4D, E and S3). Although overexpression of Ets-1 showed a modest effect on CDH1 (gene for E-cadherin, and an epithelial marker) expression (Fig. 4C), it showed a distinct increase when Ets-1 was knocked down (Fig. 4E), suggesting a reversal of EMT. Knocking down Ets-1 expression also reduced migration in the soraR cells (Fig. 4F, G). These suggested that higher expression of Ets-1 is involved in promoting EMT and other pro-oncogenic pathways to promote resistance in HCC.

### Ets-1 promotes sorafenib resistance in HCC cells

**Fig. 5:**
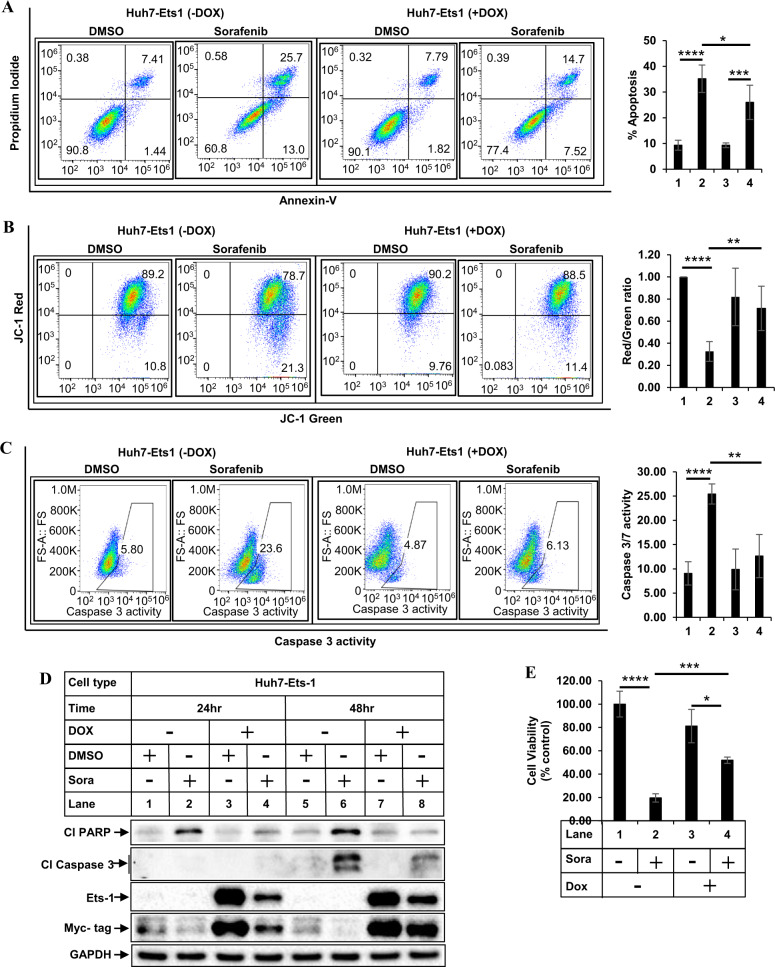
Overexpression of Ets-1 promotes sorafenib resistance. Huh7-Ets-1 WT stable cells were treated with (+) or without (−) DOX for 24 hours to induce ectopic Ets-1 expression, followed by treatment with DMSO or sorafenib (6 µM) for 48 h and flow cytometry to detect apoptosis (**A**), mitochondrial damage (**B**), or caspase 3/7 activity (**C**). The bar graphs on the right of **A**–**C** represent the degree of apoptosis, mitochondrial damage, and caspase 3/7 activity respectively. Lanes 1 and 2 were treated without DOX and lanes 3 and 4 were treated with DOX, along with DMSO (lanes 1, 3) or sorafenib (lanes 2, 4). The data represent the mean ± S.D. of at least 4–6 independent experiments. **D** Huh7-Ets-1 WT stable cells treated with (+) or without (−) DOX for 24 h followed by treatment with DMSO or sorafenib (6 µM) for an additional 24 and 48 h were analyzed by western blots. Cl PARP cleaved PARP, Cl Caspase 3 cleaved caspase 3. **E** Huh7-Ets-1 WT cells pretreated with DOX followed by treatment with DMSO (−) or 6 µM sorafenib (+) for 72 h were analyzed by MTT assay. The data represent the mean ± S.D. of three independent assays. Significant differences were determined by *t* test and indicated as: *p ≤ 0.05; **p ≤ 0.01; ***p ≤ 0.001; ****p ≤ 0.0001.

To understand the role of Ets-1 in mediating sorafenib resistance, Huh7-Ets1-WT and Hep3B-Ets1-WT cells were utilized. They were treated with sorafenib following induction of Ets-1, and examined for apoptosis, mitochondrial damage, and caspase 3/7 activity. These showed a reduction of sorafenib-mediated apoptosis (Figs. 5A and S4A, B), mitochondrial damage (Figs. 5B and S4C, D) and caspase 3/7 activity (Fig. 5C) following Ets-1 overexpression. Ets-1 overexpression also led to the reduced expression of cleaved PARP and cleaved caspase 3 in sorafenib-treated cells (Fig. 5D). Moreover, the cell viability of the Ets-1 overexpressing cells was higher as compared to the control cells (Fig. 5E). The results indicate that overexpression of Ets-1 could partially rescue the HCC cells from apoptosis induced by sorafenib.

**Fig. 6:**
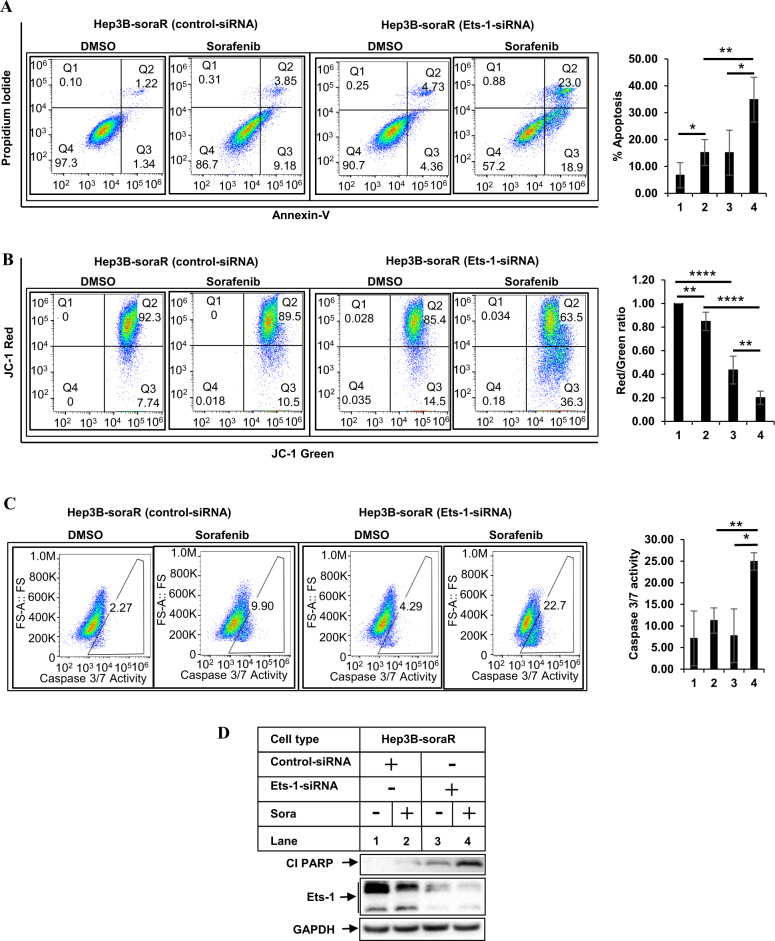
Ets-1 antagonism promotes sorafenib sensitivity in soraR cells. **A** Hep3B-soraR cells transiently transfected with control-siRNA or Ets-1-siRNA were treated with DMSO or sorafenib (6 µM) for 48 h and analyzed by flow cytometry to detect apoptosis (**A**), mitochondrial damage (**B**), or caspase 3/7 activity (**C**). The bar graphs on the right of **A**–**C** represent the degree of apoptosis, mitochondrial damage, and caspase 3/7 activity respectively. Lanes 1 and 2 were transfected with control-siRNA and lanes 3 and 4 were transfected with Ets-1-siRNA and treated with DMSO (lanes 1, 3) or sorafenib (lanes 2, 4). The data represent the mean ± S.D. of at least three independent experiments. Significant differences were determined by *t* test and indicated as: *p ≤ 0.05; **p ≤ 0.01; ****p ≤ 0.0001. **D** Hep3B-soraR cells transfected with control- or Ets-1-siRNA were treated with DMSO or sorafenib (6 µM) for 48 h and analyzed by western blots. Cl PARP cleaved PARP.

In a complementary approach, we also examined the effect of Ets-1 antagonism in reversing sorafenib resistance. To achieve this, we first utilized WP1130, which is a small molecule inhibitor of Deubiquitinase including USP9x [55, 56]. Since USP9x can prevent Ets-1 ubiquitination [57], WP1130 has been proposed as a potential pharmacological approach to target Ets-1 towards degradation [58]. Interestingly, treatment with WP1130 potently sensitized soraR cells towards apoptosis as indicated by increased PARP cleavage (Fig. S5E, F). To validate the role of Ets-1 in soraR cell survival, studies were performed following knocking down endogenous Ets-1, which showed increased sensitivity towards sorafenib as demonstrated by increased apoptosis (Figs. 6A and S5A, B), mitochondrial damage (Fig. 6B and S5C, D) and caspase 3/7 activity (Fig. 6C). This was further confirmed by the western blot results showing increased PARP cleavage with sorafenib in the soraR cells following ETS1 knockdown (Figs. 6D and S5G). These indicate that Ets-1 is involved in promoting sorafenib resistance in HCC.

### Ets-1 regulates the antioxidant pathway in sorafenib resistance

**Fig. 7:**
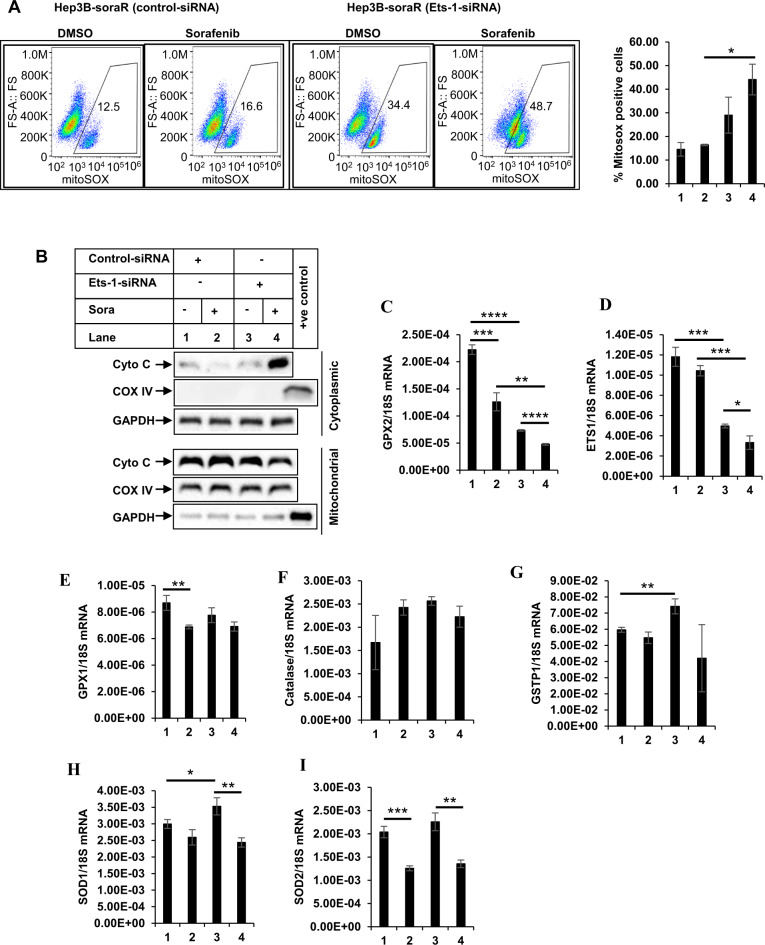
Ets-1 regulates the antioxidant pathway in sorafenib resistance. **A** Hep3B-soraR cells transiently transfected with control-siRNA or Ets-1-siRNA were treated with DMSO or sorafenib (6 µM) for 24 h and analyzed by flow cytometry to detect mROS, as described in Fig. 2E. The bar graphs on the right represent the degree of mROS induction under each condition. The data represent the mean ± S.D. of two independent experiments. **B** Hep3B-soraR cells transfected as in **A** were treated with DMSO or sorafenib for 36 h and subjected to mitochondrial and cytoplasmic fractionation and analyzed by western blots. Cyto C cytochrome C. **C**–**I** Hep3B-soraR cells transfected as in **A** were treated with DMSO or sorafenib for 24 h followed by RNA extraction and qPCR analysis of the indicated genes. The data represent the mean ± S.D. of three independent PCR reactions. In all bar graphs, lanes 1 and 2 were transfected with control-siRNA, and lanes 3 and 4 were transfected with Ets-1-siRNA and treated with DMSO (lanes 1, 3) or sorafenib (lanes 2, 4). Significant differences were determined by *t* test and indicated as: *p ≤ 0.05; **p ≤ 0.01; ***p ≤ 0.001; ****p ≤ 0.0001.

Since the soraR cells were observed to have low mROS as demonstrated in Fig. 2E, F, we next examined whether Ets-1 was involved in maintaining low mROS in sorafenib resistance. To determine this, mROS assay was performed in the soraR cells following Ets-1 knockdown. Interestingly, knocking down endogenous Ets-1 led to increased levels of mROS even in the absence of sorafenib (Fig. 7A). Knocking down Ets-1 expression also promoted the release of cytochrome c from the mitochondrial to the cytoplasmic compartment of the soraR cells upon treatment with sorafenib (Fig. 7B), suggesting a potential role of mitochondria in Ets-1-induced resistance. As mROS is often balanced by the presence of antioxidants in the cells, we also checked whether Ets-1 regulated the antioxidant genes in mediating resistance. Interestingly, although Ets-1 overexpression showed an increase in several antioxidant genes in naive cells (Fig. S6A–G), knocking down Ets-1 in the soraR cells only showed a similar reduction of GPX2 transcripts (Fig. 7C, D). The levels of other antioxidant genes were unaffected by Ets-1 knockdown (Fig. 7E-I). These strongly suggested the possibility that GPX2 might be serving as a potential downstream target of Ets-1 in mediating sorafenib resistance.

### Ets-1/GPX2 axis mediates sorafenib resistance

**Fig. 8:**
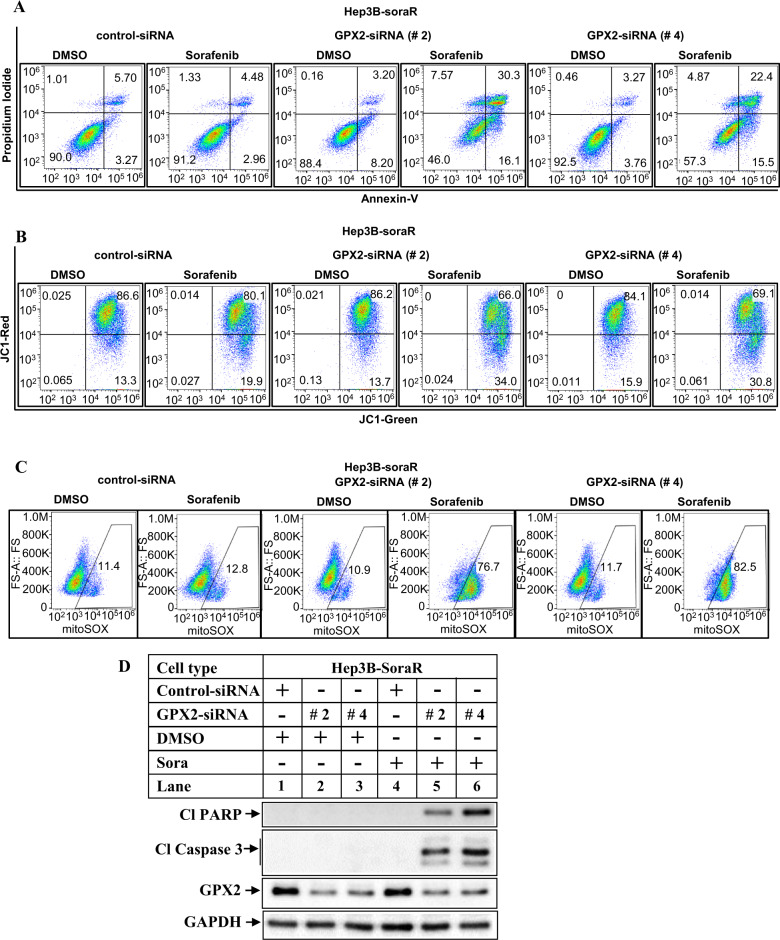
Ets-1/GPX2 axis regulates sorafenib resistance. Hep3B-soraR cells were transfected with control-siRNA or two different GPX2-siRNA (#2, #4) and treated with DMSO or sorafenib for 48 h followed by flow cytometry to detect apoptosis (**A**), mitochondrial damage (**B**), or mROS (**C**). **D** Hep3B-soraR cells transfected and treated as in **A** were analyzed by western blots with the indicated antibodies. Cl PARP cleaved PARP, Cl Caspase 3 cleaved caspase 3.

To determine whether GPX2 has any potential involvement in regulating sorafenib resistance, endogenous GPX2 was knocked down using four different siRNAs. While all of these siRNAs successfully reduced GPX2 expressions in the soraR cells (Fig. S7A, B), siRNA #2 and #4 showed maximal suppression. Interestingly, knocking down GPX2 by either siRNA #2 or #4 significantly increased sorafenib sensitivity of the soraR cells as indicated by increased apoptosis (Figs. 8A and S8A), mitochondrial damage (Figs. 8B and S8B), and mROS (Figs. 8C and S8C). Moreover, the expression of cleaved PARP and cleaved caspase 3 were highly elevated with GPX2 knockdown and sorafenib treatment in the soraR cells (Fig. 8D). These suggest that GPX2 functions as a downstream target of Ets-1 and plays a crucial role in maintaining sorafenib resistance.

## Discussion

To understand in-depth the underlying mechanisms of sorafenib resistance in HCC, in this study we identified in an unbiased way that TF Ets-1 is a potential mediator of this. Although the RT^2^ Profiler TF PCR array identified multiple genes that were induced in soraR HCCs (Fig. S2D), Ets-1 was induced specifically in all the soraR cells (Fig. 3D), confirming its generalized role in this resistance pathway. Ets-1 is known to promote cancer progression via its involvement in proliferation, EMT, invasion, angiogenesis, and drug resistance [59]. It primarily acts as a transcriptional activator, although some repressor functions have also been reported [60]. Ets-1 is also known to mediate HCC progression via induction of metastatic genes [61, 62], metabolic genes [63] and via its crosstalk with ZEB2 protein [64]. A recent paper has shown Ets-1’s participation in primary sorafenib resistance, although its involvement in acquired resistance and the downstream mechanism is still unclear, and is the focus of our study [38]. Our preliminary studies showed increased Ets-1 expression and downstream signaling in soraR HCC cells. IHC staining of human HCC TMAs showed a positive correlation of higher Ets-1 expression with HCC progression. The soraR cells used in our studies were generated by long-term culturing with sorafenib starting with a low dose and used as a model for acquired resistance. While analyzing the characteristics, the soraR cells seemed to express some stem cell characteristics as shown in Fig. 1G–K. These cells were not specifically selected to contain stem-like cells or were induced to show these phenotypes. HCC cells with acquired resistance to sorafenib have been shown earlier to have an increase in stem-like cancer cells [65]. However, since there was no significant effect of Ets-1 on sphere formation properties (data not shown), we did not focus on these pathways in the current studies. Although Ets-1 is mostly known as a prooncogenic TF, several studies have reported a paradoxical tumor-suppressive role of Ets-1 as well [66, 67, 18]. Interestingly, in a study with breast cancer cells, Ets-1 overexpression was inhibitory to in vitro soft-agar colony growth in mouse mammary tumor cells. On the other hand, in human breast cancer cells, silencing Ets-1 suppressed colony growth both in anchorage-independent assays and 3D cultures [68], suggesting a complex regulation of Ets-1 function depending on the biological environment. While the mechanisms by which Ets-1 accomplishes these opposing roles are not fully understood, one study showed that in breast cancer, Ets-1 inhibits tumorigenesis via inducing transcription of tumor-suppressor genes [66].

As reported in other resistance pathways, soraR HCC cells also showed an EMT profile, with reduced E-cadherin and increased Vimentin, ZEB2, Snail, and Slug expression levels (Fig. 1D). Modulation of Ets-1 by overexpression or knockdown produced a parallel effect on EMT, cell migration (Fig. 4), and CD44 expression (data not shown), suggesting Ets-1’s participation in various prooncogenic pathways. It is important to note that the EMT-related TFs might also play potential co-operative roles in promoting Ets1-induced resistance. For example, ZEB2 was shown to induce Ets1 transcription in HCC cells, and this crosstalk between ZEB2 and Ets-1 was responsible for the induction of TWIST and MMP9, and subsequent EMT [64, 12]. Snail was shown to mediate TGF-β-induced EMT and tumor-initiating stem-like cell properties partially, in HCC [69]. In other studies, Ets-1 was shown to regulate EMT and cancer cell invasion by promoting key EMT gene expressions such as vimentin, slug [70]. Interestingly, a Pin1 mediated Gli1-Snail-E-cadherin axis was shown to mediate regorafenib resistance in HCC [71].

To understand the role of Ets-1 on apoptosis resistance, more in-depth analyses were carried out. Overexpression of Ets-1 in naive HCC cells, reduced apoptosis, mitochondrial damage, and caspase 3/7 activation upon stimulation with sorafenib (Fig. 5), indicative of sorafenib resistance. On the other hand, pharmacological inhibition (with WP1130) or knocking down endogenous Ets-1 expression reversed these parameters and increased sorafenib sensitivity (Fig. 6, S5). To define the pathway of Ets-1-mediated resistance further, we also determined the effect of Ets-1 antagonism on mROS generation. These showed a significant increase in mROS levels following Ets-1 knockdown, suggesting that this could be the major mechanism by which Ets-1 mediates HCC resistance. An interesting recent study by Cucarull et al. suggested that antioxidants such as glutathione (GSH) can reduce the efficacy of MKIs in HCC cells by blocking mROS production [72]. Ets-1 was shown to promote higher intracellular GSH levels in resistant ovarian cancer [73] and induce transcription of xCT, an important mediator of the antioxidant pathway [74]. To understand whether Ets-1 mediated sora-resistance involved the antioxidant pathway, we also tested changes in the expression of several antioxidant genes following modulation of Ets-1 expression. To our surprise, several antioxidant genes were induced when Ets-1 was overexpressed in the naive HCC cells (Fig. S6A–G). However, only GPX2 was significantly reduced upon Ets-1 knockdown in the sora-resistant cells (Fig. 7C, D), suggesting potential compensatory mechanism(s) that might be maintaining expression of the other antioxidant genes except for GPX2, when Ets-1 was knocked down. In fact, a recent study showed the involvement of TF Nrf-2 in regulating the GPX4 pathway in sora resistance [75]. These also suggested that GPX2 could be a key downstream mediator of Ets-1-induced resistance. In fact, antagonizing GPX2 potently sensitized the soraR cells to sorafenib (Fig. 8), indicating that Ets-1/GPX2 axis is involved in the sora-resistance of HCC.

Ets-1 transcription can be induced by various growth factors (HGF, VEGF), hypoxia [59], and by Ets-1 itself [76]. Its transcriptional activity is also regulated post-translationally by MAPK pathway [77]. In fact, HGF can induce Ets-1 expression in the hepatic stellate cells [78] and HCCs [62]. Ets-1 protein stability is negatively regulated by calcium/calmodulin-dependent protein kinase II (CAMK2)-induced phosphorylation and subsequent ubiquitination-mediated degradation, but its stability is increased by c-Src and PKCα phosphorylation [79]. Thus, Src inhibitors (dasatinib, saracatinib) promote Ets-1 degradation. A recent transcriptomics study has reported the increase of Src signaling in soraR [80]. In our studies, however, Src inhibitor (dasatinib) was unable to reduce Ets-1 significantly or increase sorafenib sensitivity (data not shown). This suggested that c-Src pathway might not be a likely mechanism of Ets-1 induction in soraR cells. Since the deubiquitinase USP9X can prevent Ets-1 ubiquitination and increase protein stability [57], WP1130, an inhibitor for USP9X can serve as a potential inhibitor of Ets-1 by promoting its degradation [58]. Interestingly, treatment with WP1130 in our studies reduced Ets-1 levels and potently sensitized the soraR cells (Fig. S5E, F). An involvement of AKT in the induction of Ets-1 in sorafenib resistance has also been shown, which involves kinesin family member 14 (KIF14) [81]. In addition, HGF/c-met axis has been linked with sorafenib resistance [82, 83], and since this axis is also known to induce Ets-1 in HCCs this could be a potential mechanism by which Ets-1 is induced in sora-resistance. The precise mechanism of Ets-1 induction in soraR is still unclear. Our studies show that Ets-1 gene expression is induced in soraR, suggesting a potential involvement of transcriptional induction, although it does not rule out additional post-translational mechanisms. More mechanistic future studies are necessary to elucidate this mechanism further and identify novel signaling pathways or growth factors and cytokines that mediate Ets-1 induction in sora-resistance. Interestingly, sorafenib was able to reduce both ectopically expressed Ets-1 (Fig. 5D) and endogenous Ets-1 (Fig. 6D), via unknown mechanisms. Since it also reduced the ectopic protein, we believe it is most likely at a post-transcriptional level. One possibility is that sorafenib reduces Ets-1 protein stability via a post-translational mechanism involving ubiquitination-mediated proteasomal degradation. This is supported by the fact that WP1130, a deubiquitinase inhibitor, could reduce Ets-1 expression in the soraR cells (Fig. S5E, F). It is thus conceivable that sorafenib might regulate the expression or activity of members of the ubiquitination machinery to regulate Ets-1 post-translationally. In fact, studies have shown that sorafenib can reduce the stability of various proteins, including Mcl1 [84], HBx [85], and others [86], via proteasomal degradation pathways. The detailed mechanisms by which these are accomplished are yet to be elucidated. The other possibility is that it is regulated at the level of translation since sorafenib is also known to inhibit translation initiation and mTOR signaling in HCC cells [87]. This pathway might be similar to the translational inhibition of β-catenin that was reported by us earlier with the natural compound berberine [45]. Although studies over the last decades have revealed a wealth of information on MKIs and their connection to angiogenesis and various signaling kinases [88], in light of the recent findings [72] including ours, it will also be important to understand the detailed mechanism of MKI-resistance as well as Ets-1 induction in the context of mROS and GSH pathways. These are expected to unravel newer pathways of Ets-1 regulation as well as therapy resistance in HCC and can be utilized for future drug development.

Taken together, our studies provide a mechanism and indicate that Ets-1/GPX2 axis mediates sorafenib resistance in HCC, targeting of which in the future might help in ameliorating resistance and increasing sorafenib efficacy. Recently, other MKIs have been approved for HCC. This includes lenvatinib (as first-line alternative to sorafenib), regorafenib (as second line for those with sorafenib resistance), and cabozantinib (as second and third line) [10, 89]. Interestingly, our preliminary studies with regorafenib-resistant HCCs also show an increase in Ets-1 expression (data not shown), suggesting that these two resistance pathways might involve a similar mechanism. Whether GPX2 also serves as a downstream target of Ets-1 in regorafenib-resistant HCCs, however, remains to be determined. A recent study has also shown Ets-1’s involvement in mediating lenvatinib resistance in HCC [90], suggesting a broader role of Ets-1 in MKI resistance. These findings indicate the possibility that future targeting of Ets-1 and/or GPX2 in vivo might ameliorate HCC resistance to sorafenib as well as other MKIs. Combined targeting of these molecules along with sorafenib or other MKIs might also help in MKI dose reduction for treating primary HCC tumors. However, in the absence of specific inhibitors of Ets-1 or GPX2, direct targeting of these molecules might be challenging in the immediate future. Interestingly, a small molecule inhibitor targeting Ets-1 has been reported [91], which showed efficacy in reducing HCC proliferation and invasion. Since no other Ets-1 specific inhibitors are currently available, this inhibitor has the potential to effectively ameliorate HCC MKI resistance either as a monotherapy or sorafenib combination therapy. Another possible means of overcoming this problem will be to elucidate the upstream signaling pathways that mediate Ets-1 induction in MKI resistance. Targeting these upstream signaling pathways might be able to antagonize the Ets-1/GPX2 axis and ameliorate resistance effectively.

## Supplementary information

Supplementary Information

Reproducibility Checklist

## Data Availability

The datasets generated during and/or analyzed during the current study are available from the corresponding author on reasonable request.
