# Supplementary material for: Ets1 mediates sorafenib resistance by regulating mitochondrial ROS pathway in hepatocellular carcinoma
Source: Cell Death Dis. 2022 Jul 4;13(7):581. doi: 10.1038/s41419-022-05022-1 (PMC9253325; doi:10.1038/s41419-022-05022-1)

# Supplementary Information

#

# Ets1 mediates sorafenib-resistance by regulating mitochondrial ROS pathway in hepatocellular carcinoma

Kanchan Vishnoi^1^, Rong Ke^1^, Navin Viswakarma^1^, Piush Srivastava^1^, Sandeep Kumar^1^, Subhasis Das^1,2^, Sunil Kumar Singh^1^, Daniel R Principe ^1^, Ajay Rana^1, 2, 3^, Basabi Rana^1, 2, 3*^

^1^Department of Surgery, Division of Surgical Oncology, University of Illinois at Chicago, Chicago, IL-60612, USA

^2^University of Illinois Hospital and Health Sciences System Cancer Center, University of Illinois at Chicago, Chicago, IL-60612, USA

^3^Jesse Brown VA Medical Center, Chicago, IL-60612, USA

*To whom correspondence should be addressed: Clinical Sciences Building, MC 958, Rm. 638, University of Illinois at Chicago, 840 S. Wood Street, Chicago IL 60612, USA. Tel.: 312-996-1078; Fax: 312-996-9365: E-mail: [basrana@uic.edu](mailto:basrana@uic.edu).

**Keywords:** sorafenib-resistance, multikinase inhibitors, hepatocellular carcinoma, Ets-1, GPX2 mitochondrial ROS, apoptosis

**Running Title:** Ets-1 and GPX2 mediate sorafenib resistance

**Supplementary Table S1**

**Primers Used for qPCR analysis**

| **Target** | **Forward** | **Reverse** |
| --- | --- | --- |
| Nanog | 5’-ACCTTGGCTGCCGTCTCTGG | 5’-AGCAAAGCCTCCCAATCCCAAACA |
| Oct4 | 5’-TTTTGGTACCCCAGGCTATG | 5’-GCAGGCACCTCAGTTTGAAT |
| SOX2 | 5’-GAGCTTTGCAGGAAGTTTGC | 5’-GCAAGAAGCCTCTCCTTGAA |
| KLF4  Ets-1  E-cadherin  Vimentin  Zeb2  SNAIL  GPX2  GPX1  Catalase  GSTP1  SOD1  SOD2 | 5’-GCAGCCACCTGGCGAGTCTG  5’-AAGTCGATCTGGAGCTTTTCC  5’-CTGCCAACTGGCTGGAGATT  5’-AACTTAGGGGCGCTCTTGTC  5’-CCTGGCACAACAACGAGATTC  5’-GTTTACCTTCCAGCAGCCCT  5’-CTCACTCTGCGCTTCACCAT  5’-GAGTCTGGCTACTCTCTCGT  5’-CTCCGGAACAACAGCCTTCT  5’-GCTCTATGGGAAGGACCAGC  5’-ACTTGGGCAATGTGACTGC  5’-TCTTCAGCCTGCACTGAAGTT | 5’-CCGCCAGCGGTTATTCGGGG  5’-CAGTCGTTGCTGTTCTTTAGTG  5’-TGCTGTTCTTCACGTGCTCA  5’-CGCTGCTAGTTCTCAGTGCT  5’-AATTGCGGTCTGGATCGTGG  5’-TCCCAGATGAGCATTGGCAG  5’-TGCCCCGGAACGTATTGAAA  5’-GGATTTTGCCCTCCATGCG  5’-ATAGAATGCCCGCACCTGAG  5’-CCCGCCTCATAGTTGGTGTA  5’-GCCAATGATGCAATGGTCTCC  5’-GATGGCTTCCAGCAACTCCC |
| 18S | 5’GGCCCTGTAATTGGAATGAGTC | 5’-CCAAGATCCAACTACGAGCTT |

**Supplementary Data:**

**
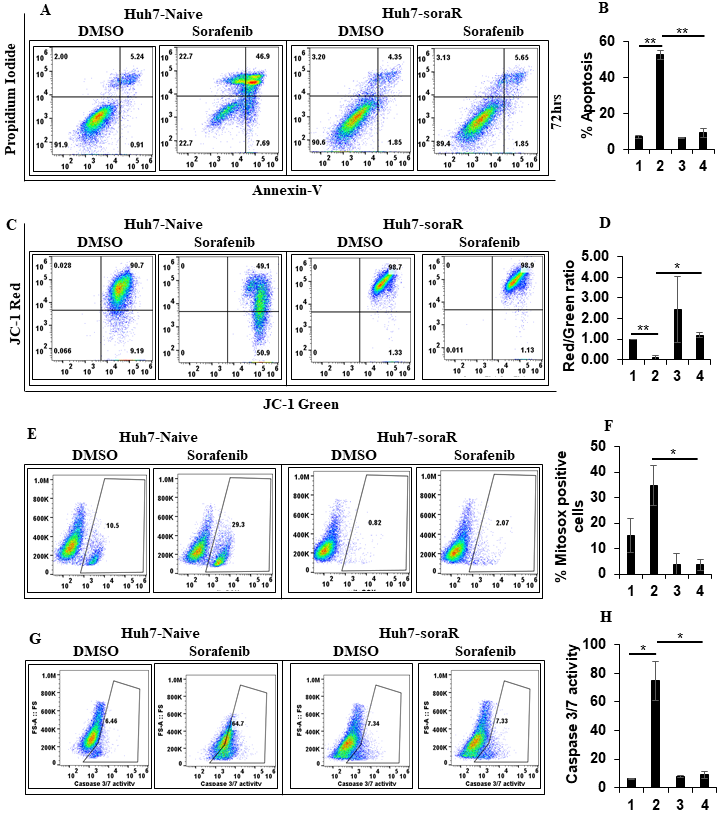
**

**Figure S1: Huh7-soraR cells are resistant to sorafenib.** Representative images of flow-cytometric detection of apoptosis **(A)**, mitochondrial damage **(C)**, or Caspase-3/7 activity **(G)** in naïve and soraR Huh7 cells treated with DMSO or sorafenib (6µM) for 72hours. The bar graphs in **(B)** represent the % of total apoptosis, in **(D)** represent the ratio of JC-1 Red/JC-1 green, and in **(H)** represent changes in caspase 3/7 activity under different treatment conditions. Representative images of flow cytometric detection of mROS in Huh7-naïve and soraR cells treated as in (A) for 24hrs are shown in **(E)**. The bar graphs on the right **(F)** represent the % of mitosox positive cells. The data (in B, D, F, H) represent the mean + S.D. of at least two independent experiments. Huh7-N cells treated with DMSO (lane 1), or sorafenib (lane 2); Huh7-SR cells treated with DMSO (lane 3), or sorafenib (lane 4). *Significant differences were determined by t-test and indicated as: *, p≤ 0.05, **, p≤ 0.01*.

**
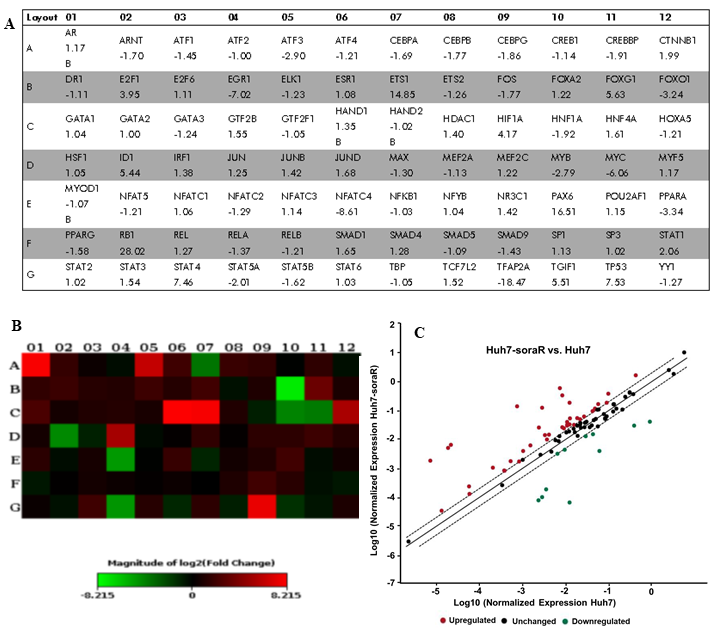
**

**
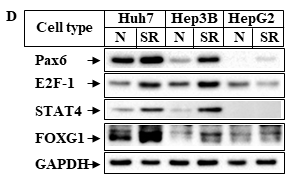
**

**Figure S2: Increased expression of Ets-1 in soraR cells. (A)** Heatmap table of fold changes in gene expression of Hep3B-soraR vs Hep3B from RT2 profiler experiment described in main Figure 3A. **(B)** Total RNA extracted from Huh7-naïve and soraR cells were subjected to cDNA synthesis and analyzed by PCR Array (PAHS-075Z), as in main Fig 3A. Figure in (B) shows the heat map with the upregulated genes marked in red and the downregulated genes marked in green. **(C)** shows the scatter plot of expression levels of 84 genes. Red dots indicate genes upregulated, the green dots indicate the genes downregulated, and the central line indicates genes that were unchanged (black dots). **(D)** Equal amounts of total protein from naïve (N) and soraR (SR) HCC cells were analyzed by western blots with the indicated antibodies.


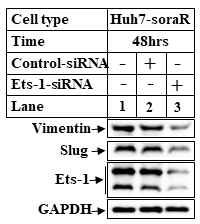


**Figure S3: Effect of Ets-1 knockdown on EMT proteins.** Huh7-soraR cells were transiently transfected with control-siRNA or Ets-1-siRNA for 48 hours and analyzed by western blots with the indicated antibodies. Lane 1 is untransfected control.


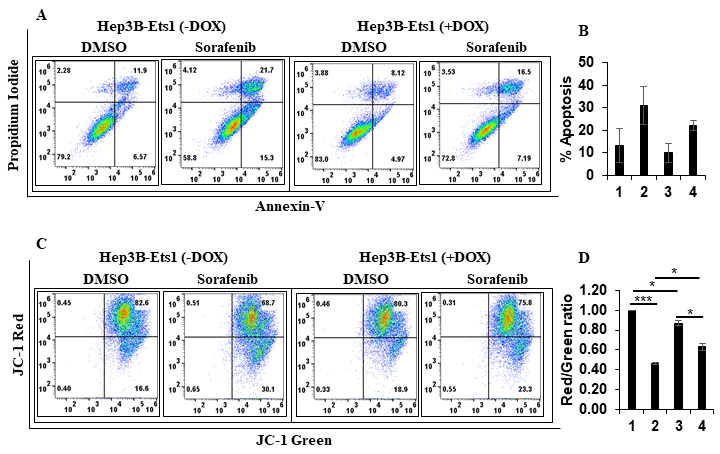


**Figure S4: Overexpression of Ets-1 antagonizes sorafenib-induced cell death.** Hep3B-Ets-1 WT stable cells were treated with (+) or without (-) DOX for 24hours to induce ectopic Ets-1 expression, followed by treatment with DMSO or sorafenib (6µM) for 24hrs and flow cytometry to detect apoptosis **(A)**, mitochondrial damage **(C)**. The bar graphs on the right represent the degree of apoptosis (B), and mitochondrial damage (D), respectively. Lanes 1 & 2 were treated without DOX and lanes 3 & 4 were treated with DOX, along with DMSO (lanes 1, 3) or sorafenib (lanes 2, 4). The data represent the mean + S.D. of at least 2 independent experiments. *Significant differences were determined by t-test and indicated as: *, p≤ 0.05; *** p≤ 0.001*.


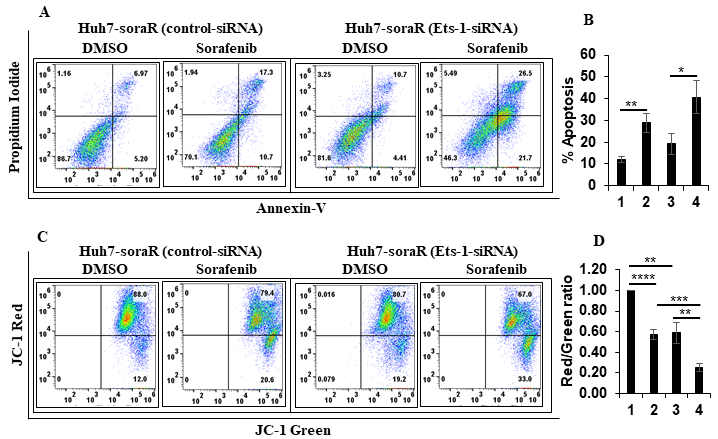


**
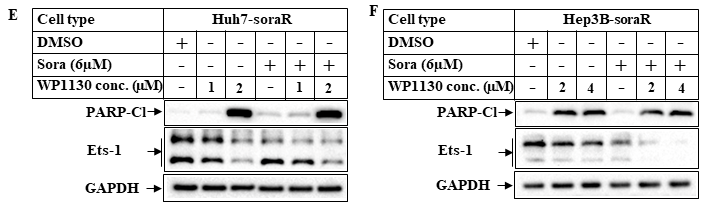
**

**
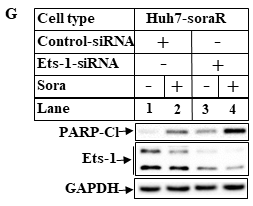
**

**Figure S5: Ets-1 antagonism increases sorafenib sensitivity**. Huh7-soraR cells transiently transfected with control- or Ets-1-siRNA were treated with DMSO or sorafenib (6µM) for 48 hrs and analyzed by flow cytometry to detect apoptosis **(A)**, mitochondrial damage **(C)**. The bar graphs on the right represent the degree of apoptosis **(B)**, and mitochondrial damage **(D)**. Lanes 1 & 2 were transfected with control-siRNA and lanes 3 & 4 were transfected with Ets-1-siRNA and treated with DMSO (lanes 1, 3) or sorafenib (lanes 2, 4). The data represent the mean + S.D. of at least 3 independent experiments. Huh7-soraR **(E)** and Hep3B-soraR **(F)** cells were treated for 24 hours with sorafenib in the presence or absence of indicated concentrations of WP1130, followed by western blot analyses. **(G)** Huh7-soraR cells transfected with control- or Ets-1-siRNA were treated with DMSO or sorafenib (6µM) for 48 hours and analyzed by western blots. PARP-Cl, PARP cleaved. *Significant differences were determined by t-test and indicated as *, p≤ 0.05; **, p≤ 0.01; *** p≤ 0.001; **** p≤ 0.0001*.

**
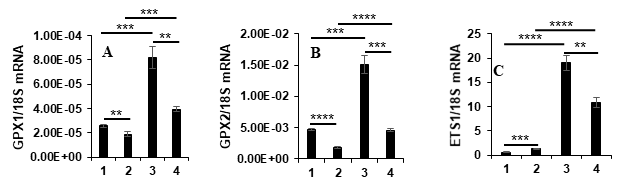
**

**
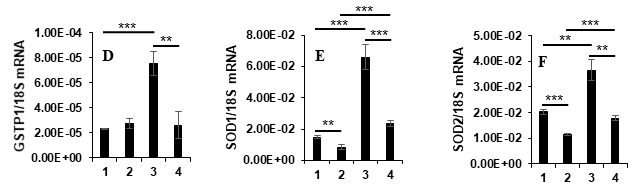
**

**
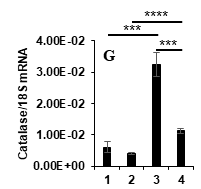
**

**Figure S6: Overexpression of Ets-1 promotes antioxidant gene expression**. Hep3B-naïve cells were transiently transfected with pLIX_403 Ets-1 vector were treated with DOX for 24 hours (to induce Ets-1), followed by treatment with DMSO or sorafenib (6µM) for an additional 24 hours. Equal amounts of RNA were analyzed by qPCR for the indicated genes **(A-G)**. In all bar graphs, lanes 1 & 2 were treated without DOX and lanes 3 & 4 were treated with DOX, along with DMSO (lanes 1, 3) or sorafenib (lanes 2, 4). The data represent the mean + S.D. of 3 independent PCR reactions. *Significant differences were determined by t-test and indicated as: **, p≤ 0.01; *** p≤ 0.001; **** p≤ 0.0001*.


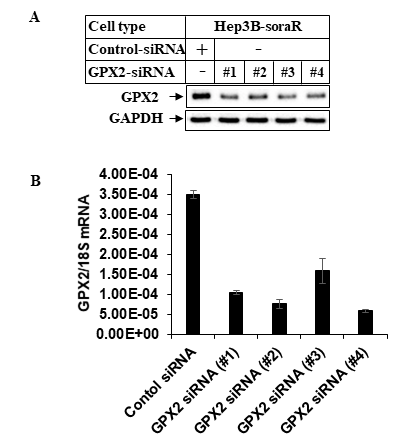


**Figure S7: Optimization of GPX2 knockdown in Hep3B-soraR cells**. Hep3B-soraR cells transiently transfected with control-siRNA or four different GPX2-siRNA for 48hrs. The degree of GPX2 knockdown was then determined by western blot **(A)** or qPCR analysis **(B)**. The data represent the mean + S.D. of 3 independent PCR reactions.

**
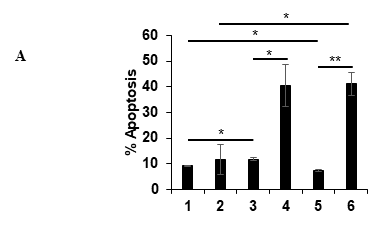
**

**
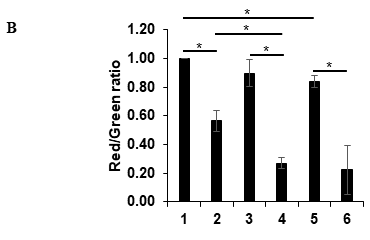
**

**
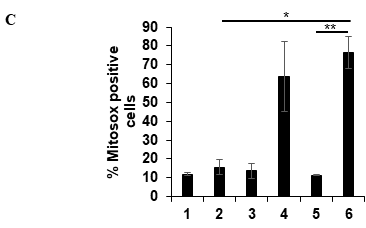
**

**Figure S8: Ets-1/GPX2 axis regulates sorafenib-resistance.** Hep3B-soraR cells were transfected with control-siRNA or two different GPX2-siRNA (#2, #4) and treated with DMSO or sorafenib as described in main fig 8 followed by flow cytometry. The bar graphs represent the degree of apoptosis **(A)**, mitochondrial damage **(B)** and mROS **(C)**, as detected in main figs 8A, B, C respectively. Lanes 1 & 2 were transfected with control-siRNA, lanes 3 & 4 with GPX2-siRNA (#2), and lanes 5 & 6 with GPX2-siRNA (#4). Treatments were done with DMSO (lanes 1, 3, 5) and with sorafenib (lanes 2, 4, 6) for 48 hrs. The data represent the mean + S.D. of at least 2 independent experiments. *Significant differences were determined by t-test and indicated as: *, p≤ 0.05; **, p≤ 0.01*.

**Supplementary Fig S9 shows Full-length uncropped blots used in this study**

**Supplementary Fig S9**

**Main Figures:**


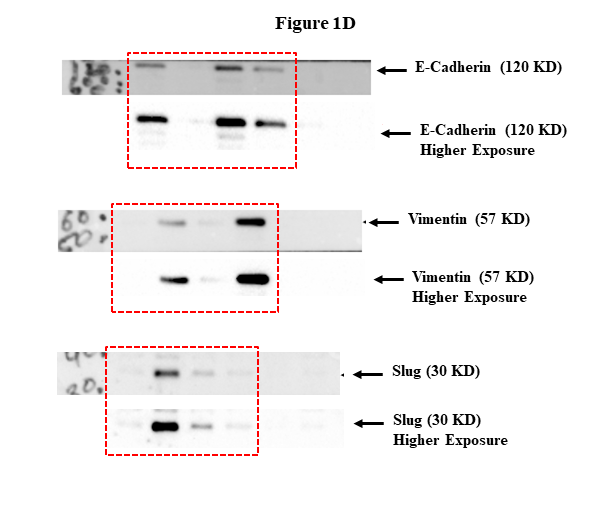


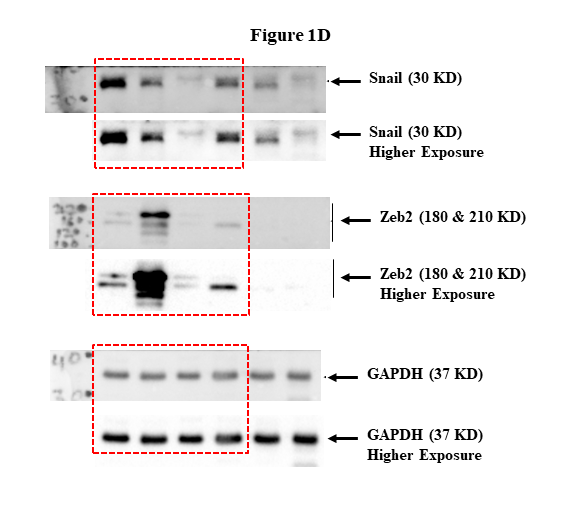


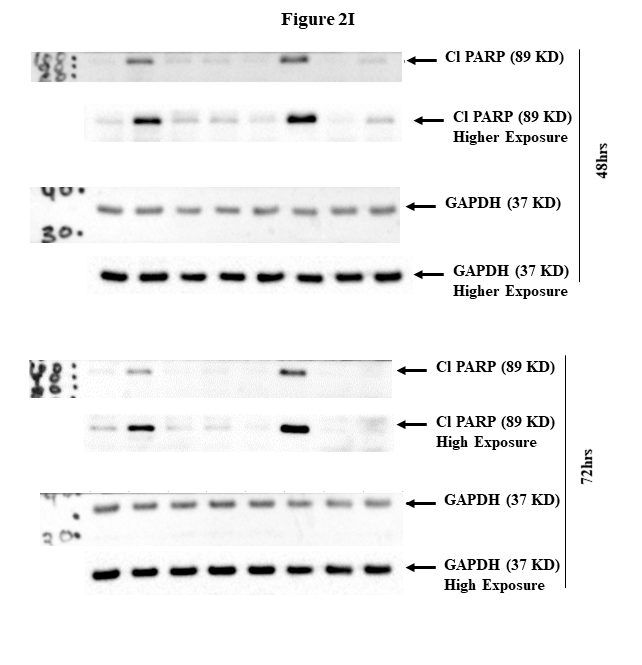


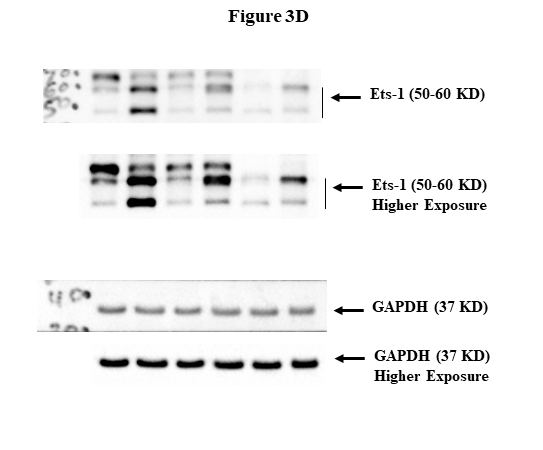


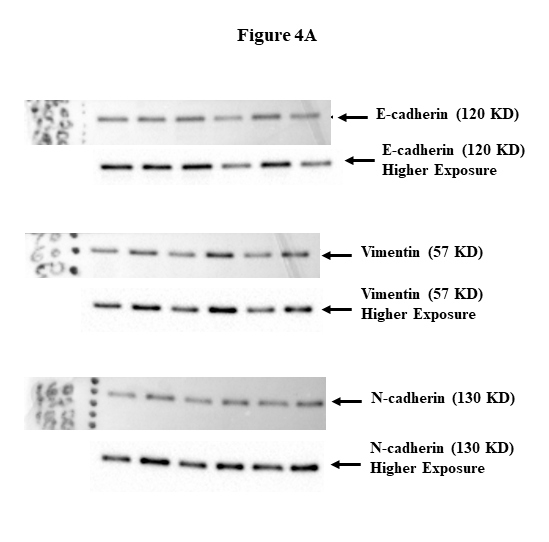


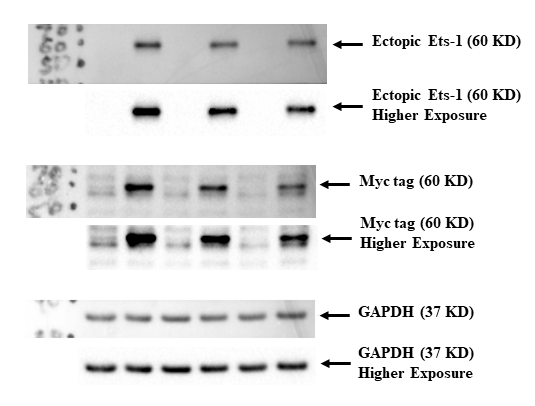


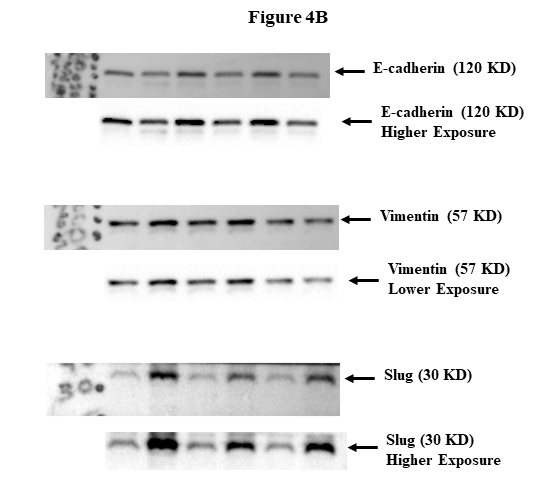


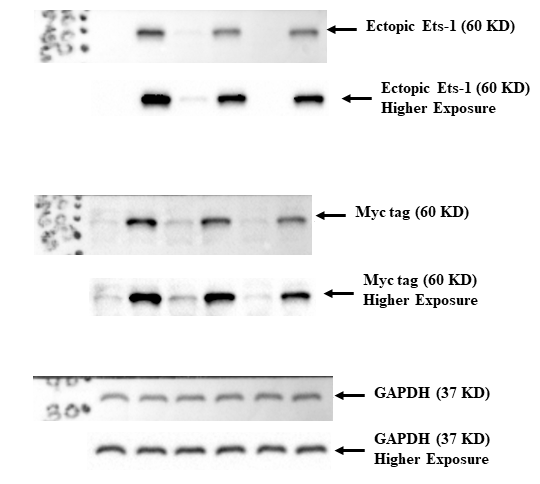


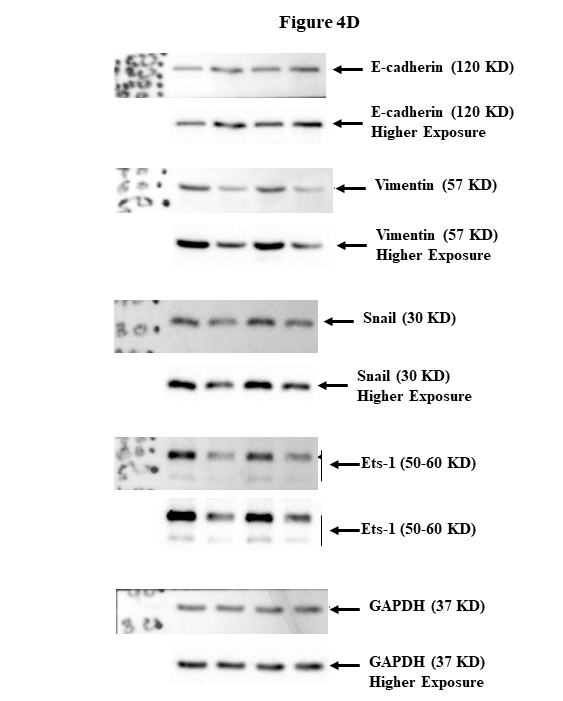


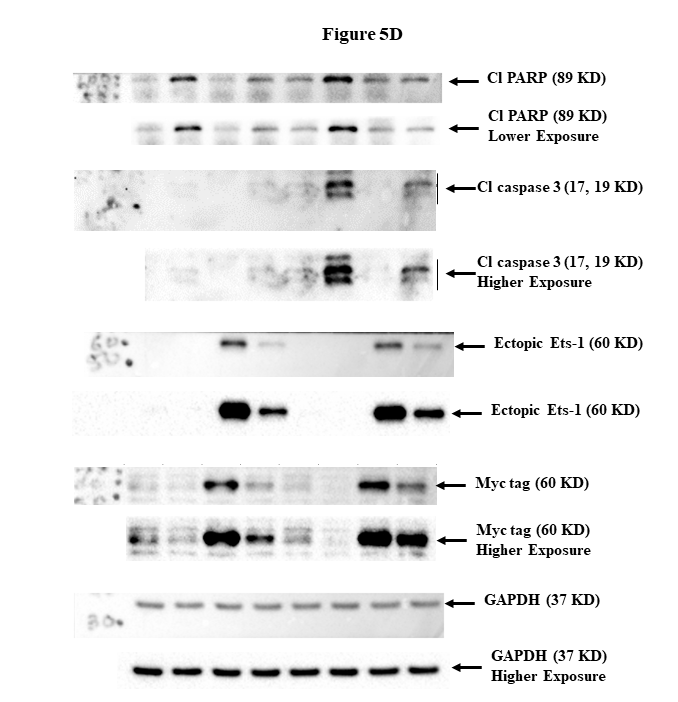


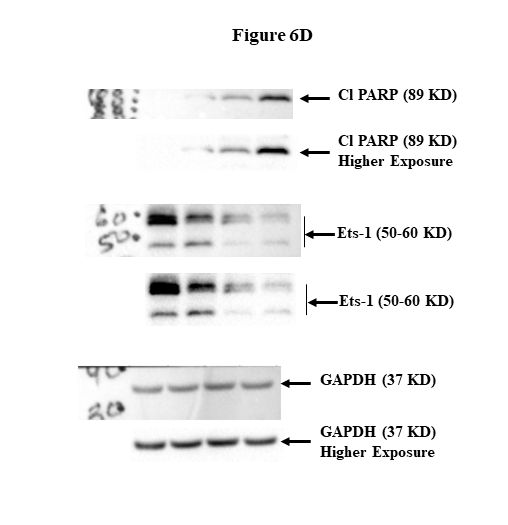


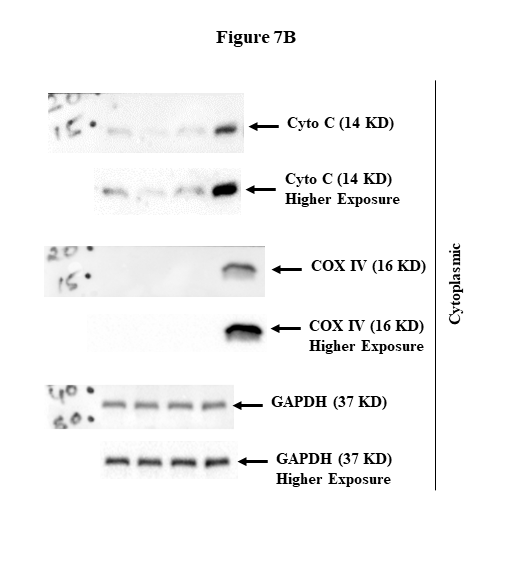


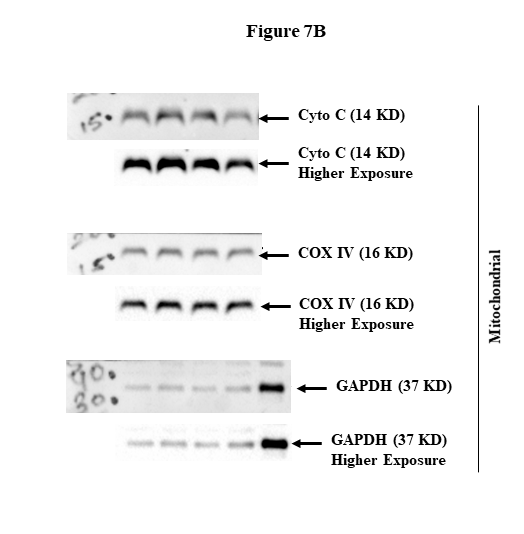


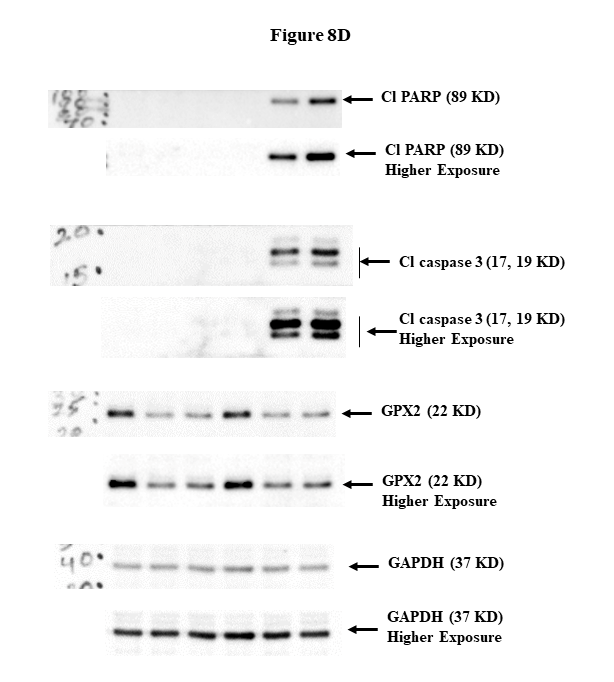

Supplement: Supplementary file 1 — Supplementary Information [file 41419_2022_5022_MOESM1_ESM.doc]
